# Association between metformin use and below-the-knee arterial calcification score in type 2 diabetic patients

**DOI:** 10.1186/s12933-017-0509-7

**Published:** 2017-02-15

**Authors:** Aurélien Mary, Agnes Hartemann, Sophie Liabeuf, Carole Elodie Aubert, Salim Kemel, Joe Elie Salem, Philippe Cluzel, Aurélie Lenglet, Ziad A. Massy, Jean-Daniel Lalau, Romuald Mentaverri, Olivier Bourron, Saïd Kamel

**Affiliations:** 1 INSERM U-1088, Pathophysiological Mechanisms and Consequences of Cardiovascular Calcifications, 80025 Amiens, France; 2 Amiens University Medical Center, Pharmacy, 80054 Amiens, France; 3 Universite de Picardie Jules Verne, UFR Pharmacie, 80025 Amiens, France; 4 Pitié Salpêtrière Hospital, Diabetology, 75005 Paris, France; 5 Universite Paris-Sorbonne, UMPC - Paris 06, 75005 Paris, Île-de-France France; 6 INSERM UMR_S 1138, Centre de recherche des Cordeliers, 75006 Paris, France; 7 Institute of Cardiometabolism and Nutrition, Paris, France; 8 Amiens University Hospital, Clinical Research Centre, Division of Clinical Pharmacology, 80054 Amiens, France; 9 Bern University Hospital, University of Bern, General Internal Medicine, 3012 Bern, Switzerland; 10 Pitié Salpêtrière Hospital, Cardiovascular and Interventional Radiology, 75005 Paris, France; 11 FRANCE2Biomedical Imaging Lab, 75006 Paris, France; 12 Pitié Salpêtrière Hospital, Pharmacology, 75005 Paris, France; 13 Pitié Salpêtrière Hospital, Clinical Investigation Center, CIC-1421, 75005 Paris, France; 14 Ambroise Paré Hospital, Nephrology, 92104 Boulogne-Billancourt, France; 15 Universite Versailles Saint-Quentin-en-Yvelines, Paris-Ile-de-France-Ouest, 78000 Versailles, France; 16 INSERM U-1018, Research Centre in Epidemiology and Population Health (CESP) Team 5, 94807 Villejuif, France; 17 Amiens University Medical Center, Endocrinology and Nutrition, 80054 Amiens, France; 18 Universite de Picardie Jules Verne, UFR Médecine, 80025 Amiens, France; 19 Amiens University Hospital, Bone and Endocrine Biology, 80054 Amiens, France; 20 Amiens University Hospital, Biochemistry, 80054 Amiens, France

**Keywords:** Oral pharmacological agents, Macrovascular disease, Imaging (MRI/PET/other), Biological statistics, Clinical science, Human, Clinical science and care (all)

## Abstract

**Background:**

Vascular calcification (VC) is common in type 2 diabetes, and is associated with cardiovascular complications. Recent preclinical data suggest that metformin inhibits VC both in vitro and in animal models. However, metformin’s effects in patients with diabetic VC have not previously been characterized. The present study investigated the association between metformin use and lower-limb arterial calcification in patients with type 2 diabetes and high cardiovascular risk.

**Methods:**

The DIACART cross-sectional cohort study included 198 patients with type 2 diabetes but without severe chronic kidney disease. Below-the-knee calcification scores were assessed by computed tomography and supplemented by colour duplex ultrasonography. Data on anti-diabetic drugs were carefully collected from the patients’ medical records and during patient interviews. Biochemical and clinical data were studied as potential confounding factors.

**Results:**

Metformin-treated patients had a significantly lower calcification score than metformin-free patients (mean ± standard deviation: 2033 ± 4514 and 4684 ± 9291, respectively; p = 0.01). A univariate analysis showed that metformin was associated with a significantly lower prevalence of severe below-the-knee arterial calcification (p = 0.02). VC was not significantly associated with the use of other antidiabetic drugs, including sulfonylureas, insulin, gliptin, and glucagon like peptide-1 analogues. A multivariate logistic regression analysis indicated that the association between metformin use and calcification score (odds ratio [95% confidence interval] = 0.33 [0.11–0.98]; p = 0.045) was independent of age, gender, tobacco use, renal function, previous cardiovascular disease, diabetes duration, neuropathy, retinopathy, HbA_1c_ levels, and inflammation.

**Conclusions:**

In patients with type 2 diabetes, metformin use was independently associated with a lower below-the-knee arterial calcification score. This association may contribute to metformin’s well-known vascular protective effect. Further prospective investigations of metformin’s potential ability to inhibit VC in patients with and without type 2 diabetes are now needed to confirm these results.

## Background

Vascular calcification (VC) corresponds to the pathological deposition of calcium-phosphate crystals in the vasculature, causing several adverse cardiovascular effects. Indeed, it is now clearly established that VC is associated with cardiovascular mortality (independently of other risks factors) in the general population and in patients with type 2 diabetes [1, 2]. Type 2 diabetes is known to induce VC, principally at the media tunica of the artery (medial calcific sclerosis) [3, 4]. Medial calcification principally occurs in patients with neuropathy [5], and tibial artery calcification contributes to peripheral artery occlusive disease—a leading risk factor for amputation in type 2 diabetes [6]. Hence, limiting or decreasing arterial wall calcification is an important therapeutic objective in patients with types 2 diabetes. However, medications specifically aimed at avoiding or delaying VC are not available.

On the cellular level, VC is an active process that involves many different stimuli and signalling pathways. Vascular smooth muscle cells (VSMCs) or circulating myeloid cells express bone-related proteins (such as Runx2, Msx2, and osteocalcin) and ultimately differentiate into osteoblast-like cells [4, 7–9]. Beyond the preponderant role of calcium and phosphate homeostasis in VC, several specific factors have been associated with increased VC in patients with type 2 diabetes, such as high glucose concentration [10], advanced glycation end products [11], increased activity of the renin angiotensin system [12], and diabetic nephropathy [13]. The involvement of these specific mechanisms suggests that medications aimed at controlling hyperglycaemia could protect against VC. Some studies have indeed reported a positive relationship between HbA_1c_ levels on one hand and the coronary artery calcium score [14–16] and peripheral vascular calcification [17] on the other.

With regard to anti-diabetic medications, recently published data have highlighted metformin’s potential ability to reduce VC. In in vitro studies, exposure of rat aortic VSMCs to metformin was associated with a significant reduction in trans differentiation and calcium deposition via the activation of AMP-activated protein kinase (AMPK) and endothelial nitric oxide synthase (eNOS) [18]. These results were confirmed in vivo in rats treated with vitamin D and nicotine, in which metformin reduced VC by restoring AMPK phosphorylation and eNOS expression [19]. Cai et al. described the underlying molecular mechanism in more detail in dual ApoE and AMPK1α1 knock-out mice; activation of AMPK1α1 in VSMCs was the main factor involved in metformin’s inhibition of atherosclerotic calcification [20].

On the basis of these preclinical findings, we hypothesized that metformin use could be associated with lower levels of VC in patients with type 2 diabetes. We therefore decided to study the association between metformin use and the below-the-knee arterial calcification score in a population of patients with type 2 diabetes.

## Research design and methods

### Study design

The protocol of the DIACART Study (for “*Diabète et Calcification Arterielle*”) has been described in detail elsewhere [21]. In brief, the study included 198 patients with type 2 diabetes attending the Cardiology and Diabetology Departments at Pitié-Salpêtrière University Hospital (Paris, France) over an 8-month period (from November 2011 to July 2012). The main inclusion criteria were (i) type 2 diabetes, and (ii) high cardiovascular risk. This risk was defined as the presence of one or more of the following factors: coronary artery disease, peripheral arterial occlusive disease, and age >50 years (for men) or >60 years (for women). The main exclusion criteria were (i) stage 4 or more chronic kidney disease, defined as a glomerular filtration rate estimated according to the Modification of Diet in Renal Disease equation (eGFR-MDRD) ≤30 ml/min [22], (ii) type 1 diabetes, (iii) a history of lower-limb angioplasty or bypass. The study was performed in accordance with the principles of the Declaration of Helsinki. The study protocol was approved by the local independent ethic committee (*Comité de Protection des Personnes* “Ile de France VI”, Paris, France). Patients were provided with comprehensive information on the study’s objectives and procedures. All patients gave their written, informed consent to participation.

### Study protocol

Clinical evaluations, laboratory blood and urine tests, multislice spiral CT scans, colour duplex ultrasonography, and an interview focused on the patient’s treatments and comorbidities were performed during a 1-day hospitalization. Additional information on anti-diabetic drugs use was extracted from the patients’ medical records. All data were analyzed independently by physicians blinded to the patients’ other results. Peripheral neuropathy was evaluated according to the Neuropathy Disability Score (with a score ≥6 considered to be abnormal). Previous cardiovascular disease was defined as a history of myocardial infarction, stroke, or any invasive procedure for coronary artery disease.

### Quantification of below-the-knee artery calcification

Below-the-knee artery calcification was assessed by scanning in the craniocaudal direction (from the patella to the ankle) with a 128-slice multi detector CT system (Somatom Definition Flash, Siemens Healthcare, Forchheim, Germany) in the absence of contrast agent. Each 3-mm cross-sectional slice was individually analyzed with Heartbeat CaScore software (Philips Healthcare, Eindhoven, The Netherlands). Each 1 mm^2^ area along below-the-knee arteries with ≥130 Hounsfield units was identified and multiplied by its density score (from 1 to 4). The sum of the weighted areas was used to calculate the calcification score, according to Agatston’s method [23]. Calcification scores for the main below-the-knee arteries (the distal popliteal, anterior tibial, posterior tibial, and peroneal arteries) were summed to give an overall calcification score.

### Quantification of peripheral arterial occlusion and medial calcific sclerosis

Colour duplex ultrasonography was used to comprehensively assess VC. Peripheral arterial occlusive disease was defined as occlusion or >70% stenosis in any artery from the abdominal tree down to the foot arteries. The severity of the stenosis was rated, with a score of 0 for no stenosis or <70% stenosis, a score of 2 for >70% stenosis, and a score of 3 for occlusion. The overall occlusion score (ranging from 0 to 39) was obtained by summing each individual artery score. Medial calcific sclerosis was also graded on the basis of ultrasound imaging, with a score of 0 point if not visible, a score of 1 if discontinuous, a score of 2 if continuous, and a score of 3 if the lumen was obstructed. Using the same method, an overall lower-limb medial calcific sclerosis score was calculated (ranging from 0 to 36).

### Laboratory tests

Fasting blood were collected for measurement of HbA_1c_, glucose, ultrasensitive C-reactive protein, interleukin 6 (IL-6), calcium, phosphate, creatinine, and urine samples for measurement of microalbuminuria.

### Statistical analysis

Data are expressed as the mean ± standard deviation (SD) and median for quantitative variables or the number (percentage) for qualitative variables. Given that the calcification scores were not normally distributed, a semi-log scale was used for graphical representations. The study population was divided into metformin-treated and -non-treated groups, depending on the current prescription. Intergroup comparisons were performed with a χ^2^ test for qualitative variables, and Student’s *T* test or the Mann–Whitney test for continuous variables. χ^2^ tests were also used to compare the frequency of metformin prescription in calcification score tertiles. For logistic regression analyses, patients were divided into two groups as a function of the severity of below-the-knee calcification (a score of 0–166 Agatston units [corresponding to the first tertile] vs. a score of >166). Univariate logistic regression analyses were used to assess the association between the below-the-knee calcification score category and metformin use. To assess the independence of this association (particularly with regard to age and renal status), we performed a multivariate logistic regression that included both variables identified in univariate analyses and relevant clinical variables. Markers of the severity of diabetes (the duration of diabetes, a history of retinopathy or neuropathy, the HbA_1c_ level, and insulin treatment) were always entered as potential factors explaining the intensity of calcification. Furthermore, the likelihood ratio technique was used to test the model’s robustness. Patients with missing data were excluded solely from analyses in which the corresponding parameter was specifically included. For all tests, the threshold for statistically significance was set to *p* ≤ 0.05. All statistical analyses were performed using SPSS software (version 18.0, SPSS Inc., Chicago, IL, USA). Graphs were created using SPSS software (version 18.0, SPSS Inc., Chicago, IL, USA) or GraphPad Prism (version 5.0, GraphPad Software, La Jolla California, USA).

## Results

### Baseline characteristics

The main clinical and biochemical characteristics for the study population as a whole and for the two subgroups are summarized in Table 1. There were a few missing data for the medial calcific sclerosis score (n = 2 patients), IL-6 (n = 1). The study included 198 patients with male/female ratio: 3.95:1; and a mean ± SD age of 64.4 ± 8.4 years. The metformin group comprised 161 patients (81.3%). The mean diabetes duration was similar in the two subgroups (around 14 years). Patients in the metformin group were significantly younger, and had a slightly higher eGFR-MDRD, less neuropathy, and lower serum IL-6. It is noteworthy that patients in the metformin group tended to have less retinopathy and a lower peripheral arterial occlusion score, although the difference was not statistically significant. Other variables did not differ significantly when comparing the two groups. Metformin was the most frequently prescribed drug, followed by sulfonylureas and related drugs (52.0%), insulin (47.5%), and gliptin or GLP-1 analogues (31.8%).

**Table 1:** Demographic characteristics of the total study population and the metformin subgroups

	Total population	Metformin-treated	Metformin-untreated	*p*-value
n	198	161	37	
Age (y)	64.4 ± 8.4 (65)	63.8 ± 8.2 (64)	66.9 ± 9 (66)	0.040
Males	158 (80%)	132 (82%)	26 (70%)	0.109
Diabetes duration (y)	14.6 ± 9.3 (13)	14.7 ± 9.1 (13)	14.2 ± 10.6 (12)	0.785
Body mass index (kg/m^2^)	29.2 ± 5.3 (28.0)	29.1 ± 5.2 (28.2)	29.3 ± 5.4 (29.4)	0.829
Previous CVD	139 (70%)	112 (70%)	27 (73%)	0.683
Hypertension	163 (82%)	131 (81%)	32 (86%)	0.462
Insulin treatment	94 (47%)	66 (41%)	28 (76%)	0.001
Neuropathy	31 (15%)	21 (13%)	10 (27%)	0.035
Retinopathy	37 (19%)	26 (6%)	11 (30%)	0.056
Current or ex-smoker	119 (60%)	99 (61%)	20 (54%)	0.405
Calcification score (AU)	2528 ± 5779 (524)	2033 ± 4514 (434)	4684 ± 9291 (1044)	0.012
MCS score	19.1 ± 10.9 (24)	18 ± 10.7 (20)	23.6 ± 10.8 (24)	0.010
Occlusion score	3.5 ± 5.3 (0)	3.2 ± 5.1 (0)	4.6 ± 6.1 (3)	0.073
eGFR-MDRD (ml/min)	76 ± 20 (76)	77 ± 20 (77)	70 ± 18 (71)	0.048
Microalbuminuria (mg/l)	166 ± 841 (23)	178 ± 924 (23)	110 ± 255 (17)	0.658
HbA_1c_ (%/mmol/mol)	7.8 ± 1.5 (7.5)/62 ± 16 (58)	7.8 ± 1.4 (7.5)/62 ± 15 (58)	8.0 ± 1.7 (7.5)/64 ± 19 (58)	0.521
Blood glucose (mmol/l)	8.2 ± 2.8 (7.8)	8.2 ± 2.8 (7.8)	7.9 ± 2.8 (7.6)	0.590
Calcium (mmol/l)	2.32 ± 0.11 (2.32)	2.32 ± 0.11 (2.32)	2.31 ± 0.14 (2.31)	0.738
Phosphate (mmol/l)	1.02 ± 0.15 (1.02)	1.02 ± 0.15 (1.02)	1.04 ± 0.17 (1.02)	0.390
us-CRP (mg/l)	2.2 ± 2.5 (1.2)	2.1 ± 2.4 (1.2)	2.7 ± 2.9 (1.5)	0.448
Interleukin-6 (pg/ml)	5.1 ± 22.2 (2.9)	3.5 ± 3.6 (2.8)	12.4 ± 50.5 (3.3)	0.033
LDL cholesterol (g/l)	1.93 ± 0.74 (1.81)	1.93 ± 0.76 (1.78)	2.04 ± 0.67 (1.89)	0.213
HDL cholesterol (g/l)	1.08 ± 0.33 (1.06)	1.06 ± 0.34 (1.01)	1.15 ± 0.28 (1.17)	0.128
Triglycerides (g/l)	1.58 ± 1.05 (1.26)	1.62 ± 1.11 (1.24)	1.42 ± 0.72 (1.42)	0.628

Data are expressed as the mean ± SD (median) for quantitative variables, and as the number (percentage) for qualitative variables

*CVD* cardiovascular disease, *AU* Agatston unit, *MCS* medial calcific sclerosis, *eGFR*-*MDRD* estimated glomerular filtration rate according to the Modification of Diet in Renal Disease equation, *HbA*_*1c*_ glycated haemoglobin, *us*-*CRP* ultrasensitive C-reactive protein, *LDL* low-density lipoprotein, *HDL* high-density lipoprotein

### Association between below-the-knee calcification scores and metformin treatment

**Fig. 1:**
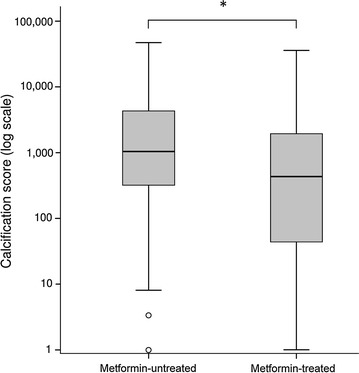
The calcification scores in patients with a current prescription of metformin (n = 161) or not (n = 37). Calcification scores for below-the-knee arteries were calculated using Agatston’s method. The median log calcification score was 2.637 in the metformin group and 3.019 in the non-metformin group (p = 0.012)

**Fig. 2:**
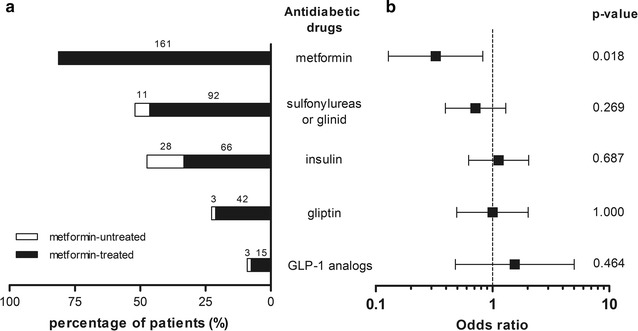
Association of antidiabetic drugs with below-the-knee arterial calcification. **a** The histogram represents the frequency of each antidiabetic drug in the cohort (n = 198). The *black bars* indicate patients treated with metformin (either alone or in combination with other medications). The *white bars* indicate patients treated with other antidiabetic drugs but not metformin. The number of patients is indicated on the different *bars*. **b** Univariate logistic regression, with a focus on pharmacological diabetic therapy (n = 198). Among the antidiabetic drugs, only the prescription of acarbose (in 4 patients) was not assessed in logistic analyses. *GLP*-*1* glucagon like peptide-1

The mean calcification score was about two-fold lower in patients with a current metformin prescription than in those without (Table 1; Fig. 1). Likewise, the medial calcific sclerosis score was significantly lower in the metformin group. Metformin was prescribed in 90.9% of the patients in the lowest calcification tertile but in only 78.8 and 74.2% of the patients in the second and the last tertiles, respectively. This difference was statistically significant when comparing all three tertiles (p = 0.04), the first and second tertiles (p = 0.05), and the first and last tertiles (p = 0.01), but not the second and last tertiles (p = 0.54). A univariate logistic regression analysis corroborated our finding that metformin prescription was associated with a lower likelihood of being in the second or last calcification tertiles (odds ratio (OR) [95% confidence interval (CI)] = 0.33 [0.13–0.83]; p = 0.02, Fig. 2). In contrast, the use of other antidiabetic drugs (including insulin), was not associated with differences in the calcification scores (Fig. 2).

### Independence of the association between metformin prescription and VC

**Fig. 3:**
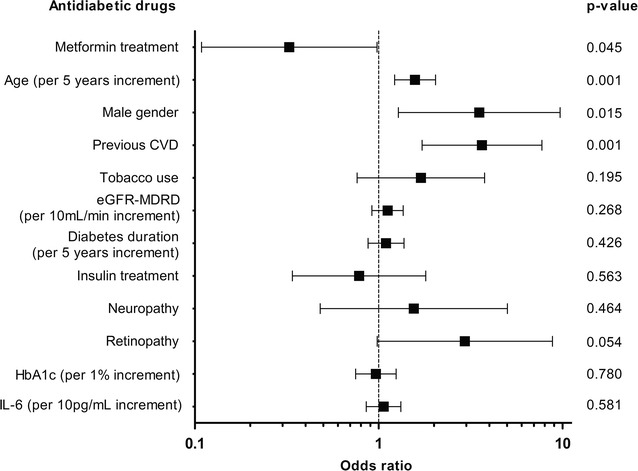
Multivariate logistic regression analysis: variables independently and significantly associated with below-the-knee arterial calcification (n = 198). The figure presents the odds ratio [95% CI] for each variable. Variables significantly associated with VC in univariate analyses and variables with relevance to diabetes were included in the multivariate model. *CVD* cardiovascular disease, *eGFR*-*MDRD* glomerular filtration rate estimated using the Modification of Diet in Renal Disease equation, *HbA*_*1c*_ glycated haemoglobin, *IL*-*6* interleukin-6

In univariate analyses, the clinical and biochemical characteristics significantly associated with the probability of having a higher calcification score than the first tertile were as follows: age (OR [95% CI] per 5 years increment = 1.44 [1.18–1.75]; *p* < 0.001), male gender (OR [95% CI] = 2.44 [1.20–4.95]; *p* = 0.014), previous cardiovascular disease (OR [95% CI] = 3.29 [1.74–6.21]; *p* < 0.001), tobacco use (OR [95% CI] = 1.87 [1.03–3.41]; *p* = 0.041), retinopathy (OR [95% CI] = 2.48 [1.03–5.99]; *p* = 0.044), and a low eGFR-MDRD (OR [95% CI] per 10 ml/min increment = 0.82 [0.71–0.96]; *p* = 0.012). In a multivariate analysis, metformin remained significantly associated with the calcification scores, independently of age, gender, previous cardiovascular disease, eGFR-MDRD, tobacco use, diabetes duration, neuropathy, retinopathy, HbA_1c_, serum IL-6, and insulin prescription (Fig. 3). When the model was simplified by applying the likelihood ratio method, metformin treatment, age, male gender, previous cardiovascular disease, and retinopathy remained significantly associated with VC (data not shown).

## Discussion

The present study is the first to demonstrate that metformin prescription in patients with type 2 diabetes is associated with lower levels of below-the-knee arterial calcification. This association was found to be independent of age, gender, renal function, diabetes duration and complications of diabetes. The majority of study participants (81.3%) were taking metformin; this reflects compliance with current guidelines on diabetes treatment and trends in the prescription of antidiabetic drugs [24]. Despite the fact that the number of patients not receiving metformin was rather small, it should be noted that the association between metformin use and lower calcification remained statistically significant in a multivariate analysis. Moreover, none of the other antidiabetic drugs taken were significantly associated with the calcification scores—suggesting that this property is specific to metformin.

The American Diabetes Association and European Association for the Study of Diabetes both recommend metformin as the first-line pharmacological treatment for type 2 diabetes [25], in view of the drug’s association with lower cardiovascular morbidity and mortality rates [26]. The results of the UK Prospective Diabetes Study showed that early intervention with metformin in patients with type 2 diabetes decreased the all-cause mortality rate by 36% (due to a decrease in macrovascular events) [27]. Metformin treatment in type 2 diabetes patients was effective in reducing the carotid intima-media thickness [28, 29] and is even associated with lower mortality rates in patients with established atherothrombosis [30]. Interestingly, metformin treatment has been shown to increase lower arterial flow in diabetes-free patients with peripheral vascular disease [31]—suggesting that the drug’s protective cardiovascular effect is not solely due to its action on blood glucose levels. The mechanisms involved in metformin-mediated cardiovascular protection have not yet been fully characterized. On the molecular level, it has been suggested that activation of AMPK might have a central role. AMPK phosphorylation by metformin improves the lipid profile [32], decreases oxidative stress [33], and protects against ischemia by maintaining ATP energy balance and increasing eNOS activity [34]. Recently published data show that metformin has a protective effect on endothelial function and angiogenesis. In streptozotocin-induced diabetic mice, metformin improved the function and increased the number of bone marrow endothelial progenitor cells (EPCs) [35]. This result has been confirmed in patients with type 1 diabetes in the MERIT study [36]. In cellular models of diabetes, metformin’s pro-angiogenic endothelial properties were found to be related to (i) AMPK/eNOS activity [35], (ii) a decrease in miR-34a levels and an increase in sirtuin1 expression [37], and (iii) secretion of vascular endothelial growth factor A together with a reduction in mRNA levels of angiogenetic inhibitors [38]. It was recently hypothesized that the EPCs’ loss of proangiogenic function in type 1 and 2 diabetes may lead to an osteogenic shift to myeloid calcifying cells, which thus links endothelial dysfunction and VC [39].

Vascular calcification is also associated with higher cardiovascular morbidity in patients with diabetes [3]. A putative metformin-related decrease in VC may thus contribute to cardiovascular protection. In below-the-knee arteries, VC favours peripheral arterial occlusive disease and thus lower-limb amputation [6, 21]. Delaying lower-limb VC through the use of metformin might decrease the risk of amputation. This is of particular interest, since a recent analysis of the UK QResearch database suggested that metformin monotherapy reduces the risk of leg amputation relative to sulfonylureas or insulin monotherapy [40].

By demonstrating an inverse relationship between metformin use and VC in patients with type 2 diabetes, our results are in line with recent in vitro and in vivo data demonstrating that metformin can inhibit VC. At present, this effect is thought to be related to AMPK activation [18–20]. Indeed, it was recently suggested that AMPKα1 activation phosphorylates STAT-1, promotes Runx2 proteasome degradation and thus decreases the differentiation of VSMCs into calcifying cells [20]. The AMPK1-related decrease in oxidative stress in endothelial cells may also reflect relevant protection against VC, since hydrogen peroxide is known to promote phenotypic switching of VSMCs by activating *RUNX2* [41]. In vitro, metformin’s AMPK phosphorylation reduces endothelial cells’ production of IL-6, a known inducer of VC [42]. Metformin treatment of patients with polycystic ovary syndrome is associated with lower serum IL-6 levels [43]. This was also the case in the present study, suggesting that IL-6 serum concentration reflects activation of AMPK by metformin. However, the fact that the association between metformin use and lower peripheral calcification was independent of IL-6 levels indicates that this was not the main mechanism.

We also found that metformin-treated patients had a higher eGFR-MDRD and were younger, less likely to be taking insulin and to have neuropathy, when compared with metformin-free patients. Given that age and eGFR-MDRD were also correlated with calcification score, the two parameters might be confounding factors that explain the intergroup differences in the peripheral calcification score. However, the results of our adjusted multivariate analysis indicate that the inverse association between VC and metformin use is independent of age and eGFR-MDRD. It has now been clearly established that chronic kidney disease (CKD) accelerates VC [13]. As severe CKD is currently a contraindication for metformin prescription (due to a risk of lactic acidosis), diabetic CKD may constitute a lost opportunity to delay VC. A potential protective effect of metformin against VC would be a further argument in favour of maintaining metformin use in patients with diabetic nephropathy, since the additional risk of lactic acidosis due to metformin remains a subject to debate [44, 45]. With a view to shedding light on this attractive prospect of metformin use in CKD, evaluation in large clinical trials is now needed.

The present study’s limitations include the cross sectional design, the relatively small number of non-metformin users, and the lack of data on metformin concentrations. A specific, prospective, randomized or case-controlled study might overcome these limitations. The present study’s strengths include its assessment of arterial calcification with a highly sensitive CT scan and ultrasonography, and a thorough analysis of the prescribed antidiabetic drugs.

## Conclusions

In conclusion, our study of patients with type 2 diabetes and high cardiovascular risk demonstrated that among antidiabetic drugs, only metformin treatment is negatively and independently associated with the severity of lower-limb artery calcification. The negative association between metformin use and VC may contribute (at least in part) to the drug’s well-known vascular protective effect. Our findings suggest that metformin use may constitute the best currently available strategy for delaying VC in type 2 diabetes, and may reinforce the justification for its first-line prescription in type 2 diabetes. Outside the field of diabetes, metformin might be a potential treatment option for other patients at risk of developing VC, such as those with CKD. These promising observations require confirmation in prospective studies.

## Abbreviations

AMPK: AMP-activated protein kinase
AU: Agatston unit
CKD: chronic kidney disease
CVD: cardiovascular disease
eGFR: estimated glomerular filtration rate
eNOS: endothelial nitric oxide synthase
EPC: endothelial progenitor cell
GLP-1: glucagon like peptide-1
HbA1c: glycated haemoglobin
HDL: high-density lipoprotein
IL-6: interleukin 6
LDL: low-density lipoprotein
us-CRP: ultrasensitive C-reactive protein
VC: vascular calcification
VSMC: vascular smooth muscle cell
